# Spray-freeze-drying aprepitant with hydroxypropyl cellulose increases nasal bioavailability

**DOI:** 10.1016/j.ijpx.2026.100551

**Published:** 2026-04-26

**Authors:** Annika Rautenberg, Paul Bühlbecker, Jan Kožák, Claire Chrétien, Yann Pellequer, Klaus Wunderling, Edmont Stoyanov, Alexander Pfeifer, Alf Lamprecht

**Affiliations:** a Department of Pharmaceutics, Institute of Pharmacy, University of Bonn, Bonn, Germany; b Université Marie et Louis Pasteur, EFS, INSERM, RIGHT (UMR 1098), F-25000 Besançon, France; c Institute of Pharmacology and Toxicology, University Hospital, University of Bonn, Bonn, Germany.; d Nisso Chemical Europe GmbH, Düsseldorf, Germany

**Keywords:** Nasal aprepitant, Porous particles, Fast absorption, Amorphous solid particles, Spray-freeze-drying, Organic freeze-drying

## Abstract

The design of free-flowing lyophilized powders by spray-freeze-drying (SFD) enables novel therapeutic applications, including nasal administration. In this study, a non-aqueous SFD approach using dimethyl sulfoxide (DMSO) as the spray solvent was investigated. Low-viscosity hydroxypropyl cellulose grades (HPC-SSL and HPC-UL) were employed as excipients to produce rapidly dissolving, porous, amorphous aprepitant powders at different drug-to-polymer ratios.

The resulting spherical particles contained amorphous aprepitant and exhibited sizes between 250 μm and 500 μm. *In vitro* dissolution at pH 7.0 showed rapid drug release, with maximum dissolution achieved after 3 min for aprepitant/HPC-UL at a 20/80 ratio, exceeding the corresponding physical mixture by 2- to 3-fold. Film-cast amorphous solid dispersions (ASDs) reached comparable drug concentrations but exhibited slower dissolution. Formulations containing HPC-SSL showed higher supersaturation levels but reduced dissolution rates. Increasing drug loading to a 40/60 ratio led to a marked delay in dissolution.

Nasal deposition *in vitro* exceeded 90% of the administered dose, attributed to the combination of low particle density (<0.1 g/cm^3^) and sufficient mechanical stability. Following nasal administration in rats, *t*_*max*_ ranged from 0.8 h to 4 h, *C*_*max*_ from 26.9 ng/ml to 64.0 ng/ml, and AUC_last_ from 125.4 ng·h/ml to 316.3 ng·h/ml, with no statistically significant differences between HPC-UL and HPC-SSL.

All SFD formulations demonstrated significantly increased nasal bioavailability compared to ASD controls. Despite similar *t*_*max*_ values, HPC-based SFD formulations enhanced aprepitant bioavailability by at least threefold. These findings underline the potential of SFD as a formulation platform for the nasal delivery of poorly water-soluble drugs.

## Highlights

• Dimethyl sulfoxide is a suitable solvent for spray-freeze-drying.
• HPC-SSL and HPC-UL stabilize aprepitant in its amorphous state.
• Nasal administration of aprepitant SFD powders results in high *in vivo* bioavailability.
• SFD particles exhibit high nasal deposition efficiency.

## Graphical abstract

**Figure:**
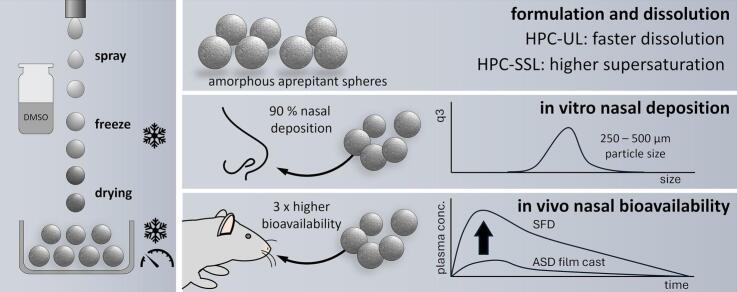

## 1 Introduction

Aprepitant is an antiemetic agent used to prevent nausea and emesis associated with chemotherapy. It belongs to the class of neurokinin-1 (NK1) receptor antagonists, and its pharmacological activity consists of blocking substance P from binding to NK1 receptors (Curran and Robinson, 2009). Since the active pharmaceutical ingredient (API) is poorly water-soluble, its dissolution under physiological conditions is decisive for bioavailability. Consequently, aprepitant is currently marketed in the form of nanocrystals for oral administration to improve its dissolution rate and increase oral bioavailability (Hargreaves et al., 2011; Shono et al., 2010; Wu et al., 2004).

In recent years, many formulation approaches have been proposed for the oral administration of aprepitant. These include soft gelatin capsules, nanocrystals (Wu et al., 2004), and amorphous solid dispersions (ASDs) of the pure drug or in combination with various excipients. These ASDs have been prepared by melt processing (Penumetcha et al., 2016), spray drying (Punčochová et al., 2015), and deep eutectic solvent-based formulations (Palmelund et al., 2021). Furthermore, a spray-freeze-dried formulation has recently been reported to improve the oral bioavailability of aprepitant (Kožák et al., 2025).

Limited attention has been paid to alternative routes of administration. In particular, aprepitant could benefit from nasal administration in its antiemetic indications, as its current clinical use is preventive. Peroral administration leads to reduced drug absorption and limits its applicability in acute nausea. In this alternative clinical scenario, aprepitant could also be used in a broader, *i.e.*, curative, therapeutic scheme. However, nasal administration of powders requires balancing two opposing parameters. On the one hand, particles must exceed a certain size threshold to avoid uncontrolled deposition in the lungs (Pozzoli et al., 2017). On the other hand, rapid drug dissolution is required to limit particle residence time on the nasal mucosa.

Spray-freeze-drying (SFD) can meet these requirements, as it produces freely flowable powders consisting of discrete, porous, spherical particles (Ali and Lamprecht, 2017; Rautenberg and Lamprecht, 2022; Wanning et al., 2015). These particles have demonstrated suitability for efficient nasal drug delivery in recent studies (Baldelli et al., 2024; Serim et al., 2021). While these results were based on aqueous SFD processes, a non-aqueous SFD technique was established to extend applicability to poorly water-soluble drugs. This approach uses tert-butanol (Kožák et al., 2026; Kožák et al., 2021; Lucas et al., 2022; Rautenberg and Lamprecht, 2024) and dimethyl sulfoxide (DMSO) as organic spray solvents (Kožák et al., 2022).

Tert-butanol and DMSO have been used as co-solvents in the freeze-drying of aqueous mixtures (Abla and Mehanna, 2025). Their use as pure solvents, providing improved solubility of the aforementioned compounds, introduces new formulation options. However, tert-butanol is flammable and has limitations for pharmaceutical use due to its toxicological profile. In contrast, DMSO has a more favorable solvent profile in terms of toxicity. It is classified as a Class 3 solvent in the ICH Q3C (R9) guideline, whereas tert-butanol is classified as Class 2 (*ICH Harmonised Guideline - Impurities: Guideline for Residual Solvents Q3C(R8)*, 2021). In addition, a DMSO content of up to 50% is considered to be acceptable in certain pharmaceutical formulations (Yoshimura et al., 2021).

DMSO has a broad solubilizing spectrum, ranging from lipophilic to hydrophilic substances (Martin et al., 1967), and enables dissolution of a wide variety of poorly water-soluble drugs (Kožák et al., 2022). However, its low vapor pressure combined with a high boiling point of approximately 190 °C complicates processing during formulation. Consequently, only a few studies have reported the use of DMSO-based freeze-drying (Den Brok et al., 2005). The integration of a spray-freezing step instead of bulk-freezing increases the available interface for solvent sublimation and enables reasonable drying times (Kožák et al., 2022).

In previous studies, two low-viscosity hydroxypropyl cellulose (HPC) grades, HPC-SSL (MW 40.000 g/mol) and HPC-UL (MW 20.000 g/mol), have demonstrated the ability to form amorphous drug formulations for various oral dosage forms (Bachmaier et al., 2021; Pöstges et al., 2022), including SFD particles for oral delivery of aprepitant (Kožák et al., 2025).

Here, we report a proof-of-concept study for the design of aprepitant SFD particles composed of the two aforementioned HPC grades for nasal administration of the poorly water-soluble API. The spraying process was initially evaluated using tert-butanol and DMSO in a comparative screening step. Subsequently, SFD particles were designed by varying the solid content up to 10% and the drug-to-polymer ratio from 20/80 to 40/60 to identify mechanical suitability for nasal administration. Moreover, the influence of formulation variables on drug dissolution was investigated, particularly regarding the impact of porosity and the ability of HPC to generate supersaturated solutions. Finally, the study was complemented by initial pharmacokinetic data in rats following nasal administration, comparing HPC-based aprepitant SFDs with film-cast aprepitant ASDs of equivalent composition.

## 2 Materials and methods

### 2.1 Materials

Aprepitant was obtained from Piramal (India). Hydroxypropyl cellulose (HPC) grades HPC-SSL (viscosity 2.0–2.92.5 mPa·s in 2% aqueous solution at 20 °C) and HPC-UL (viscosity 1.7 mPa·s in 2% aqueous solution at 20 °C) were provided by Nippon Soda Co., Ltd. (Tokyo, Japan). All other chemicals and solvents were of analytical or HPLC grade.

### 2.2 Methods

#### 2.2.1 Spray-freeze-drying

Polymer and drug solutions were prepared at room temperature under magnetic stirring by varying the aprepitant-to-HPC ratio from 20/80 to 40/60 (*w/w*) for both HPC grades (HPC-UL and HPC-SSL). The solid content of the spray solution was adjusted to either 5% or 10% (*w/v*). A spray-freeze-drying tower developed as described previously was used (Ali and Lamprecht, 2014; Kožák et al., 2022; Süverkrüp et al., 2016).

Droplets were generated using a monodisperse droplet generator (FMP, Erlangen, Germany) with a single pinhole orifice of nominal diameter 100 μm for dimethyl sulfoxide (DMSO) solutions. The droplets were frozen at approximately −100 °C. Frozen particles were collected in a glass container at the bottom of the apparatus and subsequently transferred into a freeze-dryer (Alpha 1–4 LSC Plus, Martin Christ, Germany). Freeze-drying was performed at 0.05 mbar and a condenser temperature of −50 °C under constant conditions.

#### 2.2.2 Preparation of cast HPC films

Aprepitant and either HPC-SSL or HPC-UL (20/80 or 40/60 aprepitant/HPC ratio) were dissolved in DMSO and allowed to evaporate under a fume hood at room temperature. Subsequent vacuum drying was performed for 24 h to enhance residual solvent removal.

The resulting films were milled to particles with a median diameter of approximately 260 μm and used for X-ray powder diffraction (XRPD) and dissolution testing as controls with equivalent compositions and doses.

#### 2.2.3 Contact angle

The contact angle was determined using a Krüss Drop Shape Analyzer (FM40 EasyDrop, Hamburg, Germany) by the sessile drop method. Samples were prepared using a Flexitab pneumatic hydraulic press (Röltgen GmbH & Co. KG, Solingen, Germany).

Compaction of pure substances, physical mixtures, and SFD particles was performed using 6 mm flat-face tooling and a compaction force of 7.1 kN to obtain smooth and comparable surfaces. The droplet volume was set to 5 μl. As HPC gradually dissolved, the mean contact angle measured within the first 5 s after droplet deposition was used for evaluation (*n* = 3).

#### 2.2.4 Scanning electron microscopy

The morphology of the SFD particles was analyzed using a Hitachi SU 3500 scanning electron microscope (SEM, Hitachi, Tokyo, Japan) at an acceleration voltage of 3 kV. Samples were sputter-coated with gold using a Polaron SC7640 sputter coater (Quorum Technologies Ltd., Newhaven, UK) under an argon atmosphere.

#### 2.2.5 Bulk density, particle size, and specific surface area

Bulk density was determined volumetrically by filling a graduated cylinder with loosely poured powder without compaction. The occupied volume was recorded, and bulk density was calculated as the ratio of mass to volume (*n* = 3).

Particle size distribution was measured using a Camsizer X2 dynamic image analysis system (Retsch Technology GmbH, Haan, Germany) in free-fall mode. The minimum diameter was used for particle size evaluation, and particle roundness was assessed using sphericity. Sphericity is defined as the ratio of the circumference of a circle with the same diameter as the measured particle to the circumference of the particle. Span values were calculated using the equation: SPAN = (D₉₀ − D₁₀) / D₅₀, where D₁₀, D₅₀, and D₉₀ represent the particle diameters at 10%, 50%, and 90% cumulative volume, respectively.

Specific surface area was determined by nitrogen adsorption using a Belsorp MiniX (Retsch Microtrac). Approximately 1.8 cm^3^ of powder was placed in the sample cell, and the mass was recorded. Nitrogen adsorption was measured at 11 points within a relative pressure range of 0.05–0.3 (*p/p₀*).

Free space correction was performed using advanced free space measurement mode. The specific surface area was calculated using BELMaster software (Version 7.4.3.1) according to the Brunauer-Emmett-Teller (BET) method. All samples were measured in triplicate (*n* = 3).

#### 2.2.6 Amorphous state and long-term stability

SFD powders were stored for 12 months at 25 °C and 60% relative humidity. Crystallinity was assessed by X-ray diffraction (XRD) using an X'Pert PRO diffractometer (PANalytical, Almelo, Netherlands) in reflection mode with CuKα₁ radiation at 45 kV and 40 mA.

Measurements were performed over a 2θ range of 4–50° with a step size of 0.017°. Samples were considered amorphous if a broad halo without sharp diffraction peaks was observed. Characteristic crystalline peaks of pure aprepitant served as reference.

#### 2.2.7 *In vitro* dissolution

Dissolution testing was performed using a paddle apparatus at 50 rpm in 100 ml of 0.01 M phosphate buffer (pH 7.0) containing 0.1% polysorbate 20 at 37 °C. Neutral pH conditions were selected to mimic nasal or sublingual administration.

Non-sink conditions were ensured by using approximately four times the saturation solubility of aprepitant. This corresponded to 4 mg of aprepitant (20 mg SFD particles at 20% loading; 10 mg at 40% loading).

Samples were withdrawn at predefined time points, filtered through 0.2 μm cellulose acetate filters, and analyzed by HPLC (Section 2.2.8).

Saturation solubility was determined by incubating aprepitant or physical mixtures with HPC-UL or HPC-SSL (20/80 and 40/60 ratios) for 72 h at 37 °C under agitation. The polymer-to-medium ratio matched dissolution conditions.

#### 2.2.8 High-performance liquid chromatography and mass spectrometry

During *in vitro* studies, aprepitant concentration was analyzed using a Waters Alliance high performance liquid chromatography (HPLC, Waters Corporation, Milford, USA) 2695 system with a Waters 996 photodiode array detector and a reverse-phase C-18 column (LiChrospher 100 RP-18, 5 μm, 4.6 × 250 mm; Merck, Darmstadt, Germany). The mobile phase consisted of 70:30 acetonitrile:water (*v/v*) at a flow rate of 1.0 ml/min. Detection was performed at 211 nm, and the limit of quantification (LOQ) was 0.75 μg/ml.

For plasma analysis, samples were extracted with acetonitrile and 0.1% formic acid in water containing a 100 ng/ml aprepitant-D4 (internal standard, IS). Samples were centrifuged at 20.000 ×*g* for 20 min at 4 °C, filtered, and analyzed using an Orbitrap Exploris120 mass spectrometer (MS, Thermo Fisher Scientific, Waltham, USA) coupled to a VanquishFlex HPLC (Thermo Fisher Scientific, Waltham, USA).

Separation was achieved using a Cortecs UPLC C18+ column (1.6 μm, 2.1 × 150 mm, Waters Corporation, Milford, USA). The mobile phase consisted of water +0.1% formic acid (A) and acetonitrile +0.1% formic acid (B) at 0.25 ml/min. The gradient was: 5% B (0–2.0 min), 5–100% B (2.0–10.0 min), 100% B (10.0–12.0 min), 100–5% B (12.0–12.01 min), and 5% B (12.01–22.0 min).

The retention time of aprepitant and aprepitant-D4 was 9.2 min. Injection volume was 1 μl. The mass spectrometer was operated in positive ion mode with MS1 resolution of 120.000. Aprepitant was detected at *m/z* 535.1564 [M + H]^+^ and aprepitant-D4 at *m/z* 539.1815 [M + H]^+^. Quantification was performed using an external calibration curve normalized to IS response in TraceFinder 5.1 (Thermo Fisher Scientific, Waltham, USA).

#### 2.2.9 Aerodynamic properties and particle deposition

Aerodynamic properties were evaluated using a Next Generation Cascade Impactor (NGI, Copley Scientific, Nottingham, UK) with a 1 l nasal extension chamber. Powder was administered using a custom nasal device at an airflow rate of 15 l/min (Serim et al., 2021).

The chamber was coated with glycerol to simulate the epithelial surface. After deposition, components were rinsed with a 1:1 (*v/v*) acetonitrile-water solution. Drug content was quantified by HPLC (Section 2.2.8).

For each formulation, calibration was carried out using a reference sample of the identical SFD formulation (matrix-matched calibration). This approach accounts for minor variations in actual drug loading after spray-freeze-drying (*e.g.*, due to weighing inaccuracies or residual solvent), thereby preventing systematic over- or underestimation of the deposited fraction. Deposition was expressed as a percentage of the total recovered amount. Measurements were performed in triplicate (*n* = 3).

#### 2.2.10 Pharmacokinetic studies in rats

Animal experiments were conducted in accordance with Directive 2010/63/EU. Studies were performed at the University of Franche-Comté (Besançon, France) under approval APAFIS#28241–2,020,111,616,123,922 v2. Sprague Dawley male rats (approximately 200 g, *n* = 4) were used.

SFD particles were administered nasally *via* a custom-made nasal device using a dose of 8 mg/kg aprepitant (5 mg or 10 mg of SFD particles per rat for the 5% and 10 formulations respectively). Blood samples were collected from the caudal vein at 0, 0.25, 0.5, 1, 2, 4, 8, and 24 h. Samples were mixed with citrate, centrifuged at 1.500 ×*g*, and plasma was stored at −20 °C.

#### 2.2.11 Statistical analysis

Pharmacokinetic parameters were analyzed using the Kruskal-Wallis test followed by Dunn's *post hoc* test. Statistical evaluation for contact angles, BET and nasal deposition was performed using one-way analysis of variance (ANOVA), followed by Tukey's test for multiple comparisons. Significance levels were set at *p* < 0.05 (*), with *n* = 3 per formulation.

## 3 Results

**Fig. 1:**
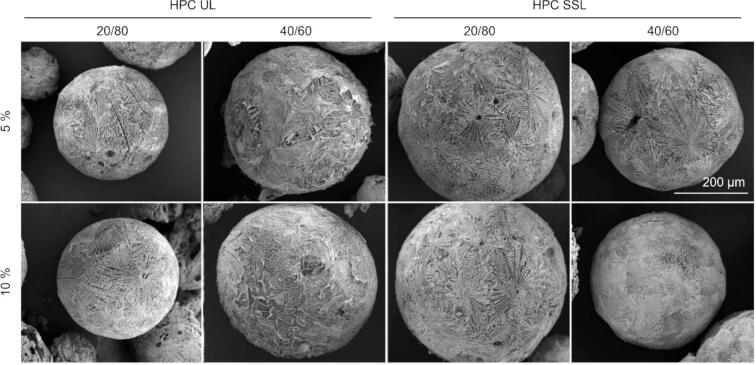
SEM images of SFD particles prepared from HPC-UL or HPC-SSL at drug-to-polymer ratios of 20/80 and 40/60, and initial spray solution concentrations of 5% and 10% (*w/v*). The scale bar applies to all images.

All SFD formulations yielded powders composed of spherical particles with low densities. Bulk density values were 0.11 ± 0.02 g/cm^3^ and 0.05 ± 0.01 g/cm^3^ for formulations with 10% and 5% solid content, respectively. Particle morphology observed by SEM was comparable across all formulations, regardless of drug-to-polymer ratio, HPC grade, or solid content (Fig. 1). High sphericity values resulted in favorable handling behavior, typically associated with good flow properties.

**Fig. 2:**
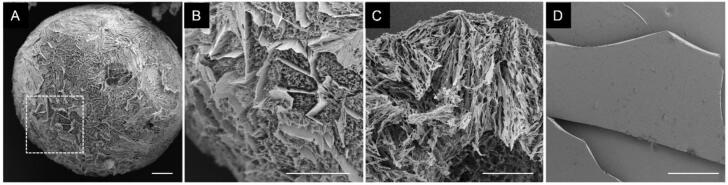
SEM images illustrating the high specific surface area of SFD particles (A-C) compared to a film-cast formulation (D). (A) shows an intact particle, (B) a magnified view of the particle surface (dotted square in A), and (C) the internal structure of a fractured particle. The scale bar represents 50 μm.

HPC acted as a mechanical stabilizer of the particle matrix. Cross-sectional images (Fig. 2C) showed distorted lamellar structures and irregular fracture surfaces rather than sharp brittle cleavage planes. The particles deformed during sectioning, indicating ductile behavior of the polymeric matrix. This reduced brittleness likely contributed to preservation of the porous architecture during handling and transport.

**Fig. 3:**
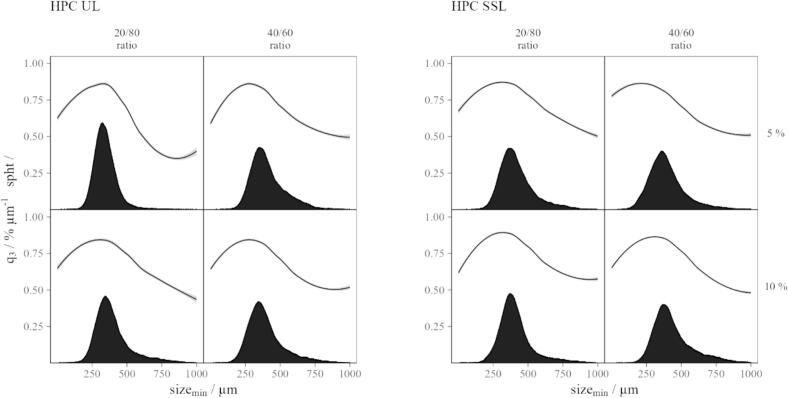
Particle size distributions and sphericity values of the SFD powders.

Median particle diameters ranged from 337 μm to 408 μm. Particle size distributions were monomodal, with SPAN values below 0.86 and average sphericity values above 0.77 (Fig. 3).

**Fig. 4:**
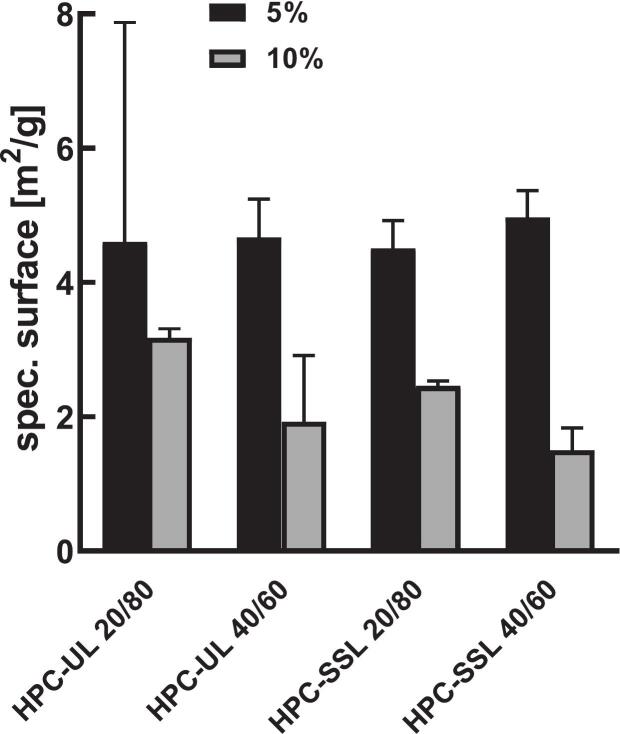
Specific surface area of the different SFD formulations determined by Brunauer-Emmett-Teller (BET) analysis. Data are presented as mean ± standard deviation (SD) (*n* = 3).

The formulations exhibited highly porous, multilamellar surface structures, as shown by high-magnification SEM images of particle surfaces (Fig. 2A,B) and cross-sections (Fig. 2C). This porosity was confirmed by large specific surface areas determined by BET analysis (Fig. 4).

Initial solid content significantly affected the specific surface area (*p* < 0.05). Formulations with 10% solid content showed a reduction of approximately 2.8 m^2^/g compared to 5% formulations. Polymer grade (HPC-UL *vs.* HPC-SSL) had no significant effect when comparing respective formulations.

All SFD formulations were XRD-amorphous after preparation, as reported previously (Kožák et al., 2025). Drug-to-polymer ratios of 40/60 were stable for 3 months at 25 °C, whereas 20/80 ratios remained stable for 12 months at 25 °C.

**Fig. 5:**
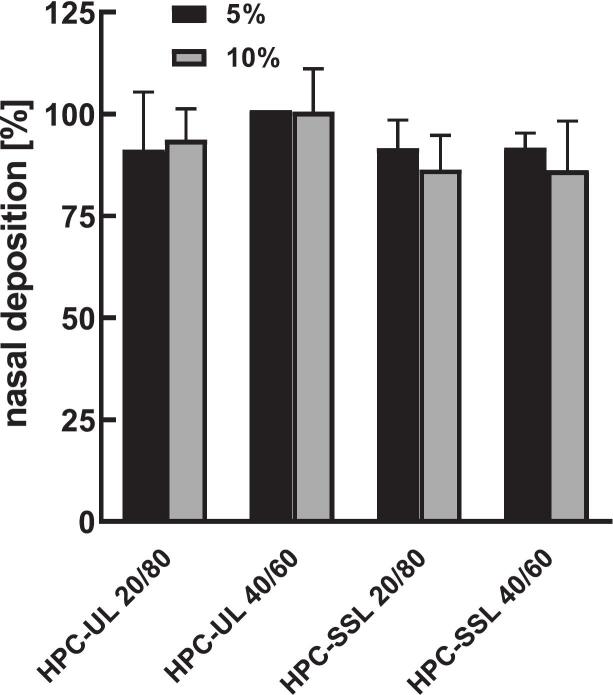
Nasal deposition measured using a Next Generation Cascade Impactor (NGI) with a nasal extension chamber. Data are presented as mean ± SD (*n* = 3).

To evaluate mechanical stability and suitability for nasal delivery, NGI experiments were performed using a nasal extension chamber. Nasal deposition exceeded 84% of the total dose, with several measurements approaching 100% (Fig. 5).

Formulations with 5% solid content were easily administered with a single actuation, resulting in consistently high deposition. In contrast, 10% formulations often required multiple actuations due to partial blockage of the 1 mm applicator orifice. This observation corresponded to slightly higher particle sizes, with average D_90_ values of 543 ± 70 μm and 588 ± 37 μm for 5% and 10% formulations, respectively. High standard deviations in some measurements were attributed to these blockages. Particles adhered strongly to the nasal chamber surface, confirming the adhesive properties of HPC.

The saturation solubility of pure aprepitant in the dissolution medium was 9.4 ± 0.1 μg/ml. HPC-UL showed no significant effect (9.3 ± 0.3 μg/ml), whereas HPC-SSL slightly increased solubility (10.0 ± 0.3 μg/ml).

**Fig. 6:**
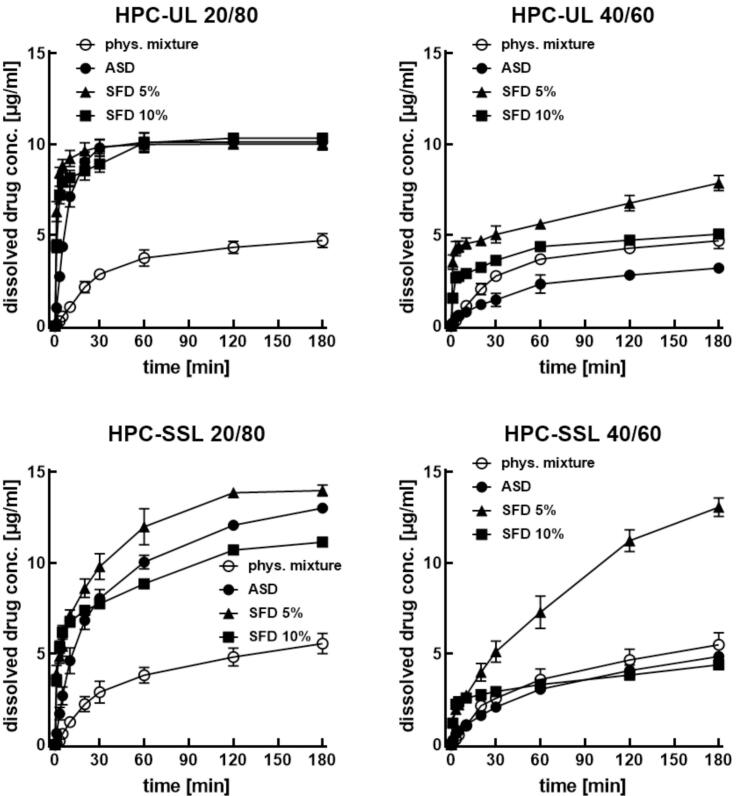
*In vitro* dissolution of HPC-based SFD particles in phosphate buffer (pH 7.0) containing 0.1% polysorbate 20. The total amount of aprepitant was kept constant at 4 mg: 20 mg SFD particles for 20% loading and 10 mg SFD particles for 40% loading. Data are presented as mean ± SD (*n* = 3).

All SFD formulations exhibited faster dissolution than crystalline aprepitant in physical mixtures (Fig. 6). Several trends were observed: faster dissolution for HPC-UL compared to HPC-SSL, faster dissolution for 20/80 compared to 40/60 ratios, and slower dissolution with increasing solid content (5% to 10%).

Overall, dissolution rates followed the order: physical mixtures < film-cast formulations < SFD (10% solid content) < SFD (5% solid content).

Considering the full dissolution profile, these trends partially changed. At a 20/80 ratio, both SFDs and amorphous solid dispersions (ASDs) achieved higher dissolved concentrations than physical mixtures. At a 40/60 ratio, SFDs with 10% solid content did not sustain enhanced dissolution and showed kinetics similar to physical mixtures (Fig. 6).

Film-cast ASDs exhibited reduced dissolution rates, particularly for HPC-UL. These differences correlated with crystallinity: at a 40/60 ratio, film-cast samples contained recrystallized aprepitant, with sharper diffraction peaks observed for HPC-UL (Kožák et al., 2025).

**Fig. 7:**
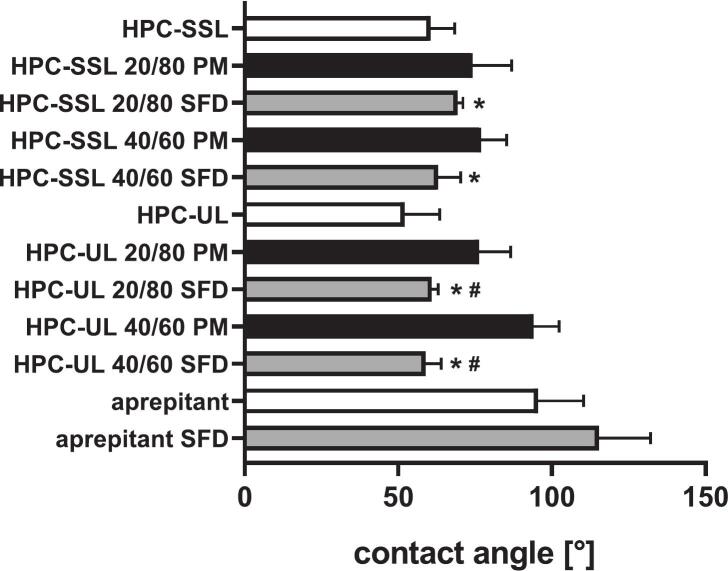
Contact angle measurements of compressed neat materials (white) and aprepitant/HPC mixtures prepared as physical mixtures (PM, black) or corresponding SFD formulations (grey), using distilled water. Data are presented as mean ± SD (*n* = 15). *p* < 0.05 compared to coarse aprepitant and aprepitant SFDs; # *p* < 0.05 compared to the respective physical mixture.

Contact angle measurements were used to assess wettability. Only minor differences were observed between formulations. A significant increase in contact angle occurred only when compared to pure aprepitant and between HPC-UL physical mixtures and corresponding SFD formulations (Fig. 7). Drug-to-polymer ratio had no significant effect, and only a slight reduction was observed for HPC-UL compared to HPC-SSL. Similar results were obtained using dissolution medium instead of distilled water (data not shown).

**Fig. 8:**
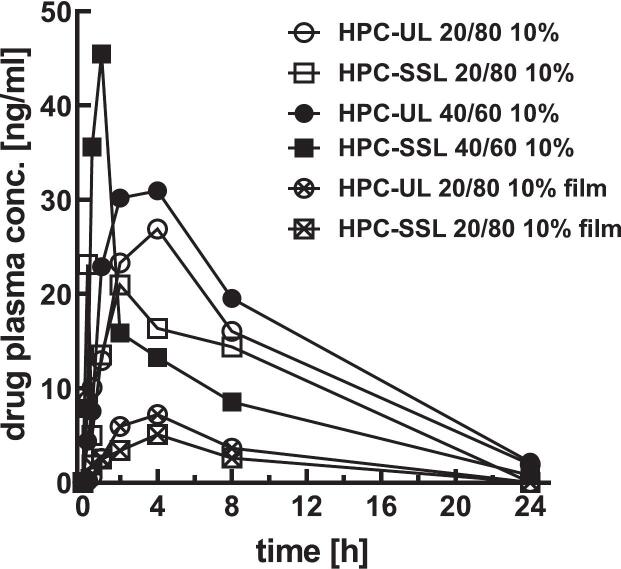
*In vivo* pharmacokinetics in rats after nasal administration of aprepitant SFD formulations (20/80, HPC-UL or HPC-SSL, 10% solid content) compared to film-cast ASD formulations of identical composition. Data are presented as mean (*n* = 3); error bars are omitted for clarity.

**Fig. 9:**
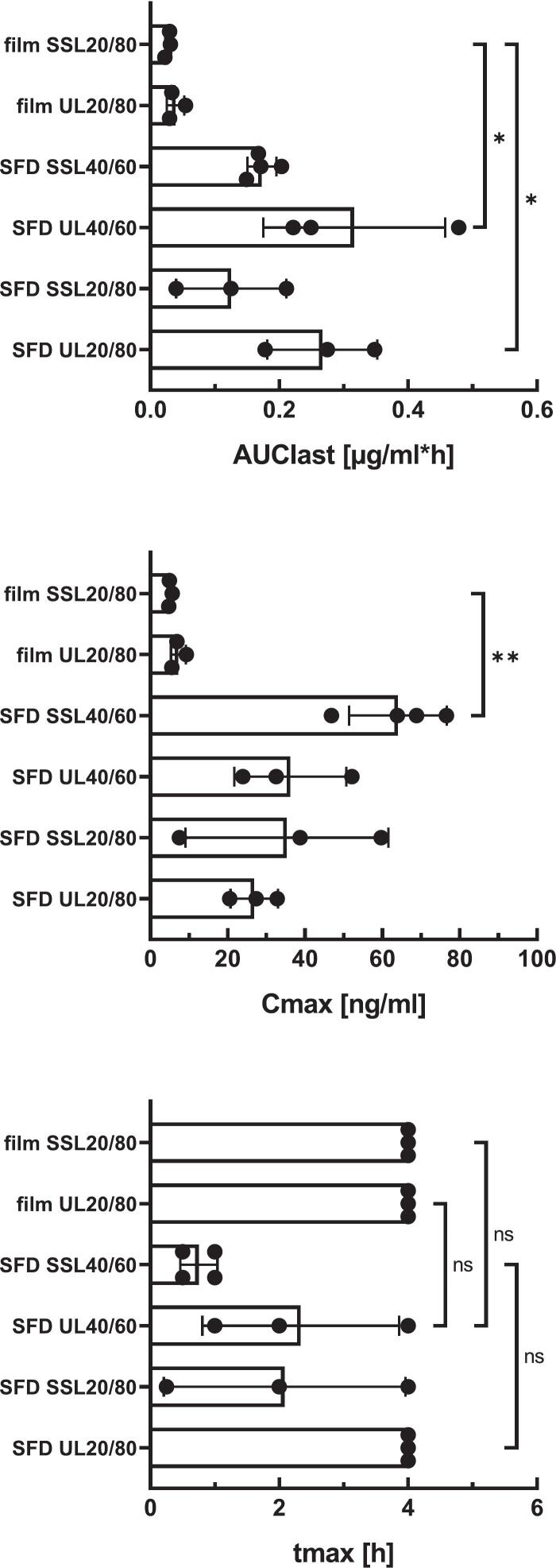
Key pharmacokinetic parameters following nasal administration of SFD formulations (10% solid content) compared to film-cast aprepitant formulations. Significant differences in AUCₗₐₛₜ (*p* < 0.05) were observed for HPC-UL SFDs (20/80 and 40/60) compared to film-cast HPC-SSL, whereas comparisons with film-cast HPC-UL were not significant (*p* = 0.070 and *p* = 0.135).

Formulations with 10% solid content were selected for *in vivo* evaluation due to their higher drug load, which is relevant given the limited nasal dosing volume in rodents (Gross et al., 1982). Formulations with 20/80 and 40/60 ratios and corresponding film-cast ASDs were tested (Fig. 8, Fig. 9).

Compared to film-cast ASDs (20/80), SFD formulations resulted in a 7–8-fold increase in AUC_last_ for HPC-UL and a 4–5-fold increase for HPC-SSL. No significant differences in *t*_*max*_ were observed, although a slightly faster onset was noted for HPC-SSL (Fig. 8, Fig. 9). This effect was also visible in individual pharmacokinetic profiles (Fig. S1).

## 4 Discussion

The SFD powders exhibited high mechanical stability despite their low density, combined with favorable flow characteristics inferred from particle morphology and handling behavior. This is in line with previous reports for SFD particles based on aqueous and organic solvents (Kožák et al., 2022; Lucas et al., 2022; Rautenberg et al., 2026; Rautenberg and Lamprecht, 2025). The mechanical stability of the particles is considered a prerequisite for efficient and selective nasal administration of powders, in order to avoid deposition of powder fragments in the deeper respiratory tract.

A critical consideration for non-aqueous spray freeze-drying is the choice of solvent and the residual solvent levels in the final dry powder. DMSO was selected in this study in part because it is categorized as a Class 3 solvent with low toxic potential under the ICH Q3C (R9) guideline for residual solvents (*ICH Harmonised Guideline - Impurities: Guideline for Residual Solvents Q3C(R8)*, 2021), where allowable daily exposures are high and no health-based exposure limits are specified at typical trace levels for pharmaceutical products. DMSO also exhibits favorable physical properties, including a high flash point (≈ 95 °C)(Gallardo-Villagrán et al., 2022), compared with alternatives such as tert-butanol (≈ 17.5 °C) (Lee et al., 2021), thereby reducing processing hazards and flame risks.

Upon solvent removal during SFD, residual DMSO levels in the resulting powders were quantified and found to be substantially below the permitted daily exposure threshold of < 50 mg/day for Class 3 solvents (Kožák et al., 2025), mitigating toxicological concerns for nasal administration. This consideration is particularly important for intranasal applications, where mucosal irritation and systemic absorption are relevant. Previous studies have shown that trace levels of Class 3 solvents such as DMSO do not elicit significant local toxicity when present below regulatory limits (Kollerup Madsen et al., 2018; Maher et al., 2022). Furthermore, DMSO's pharmacokinetic profile and extensive use in clinical formulations support its suitability as a processing solvent when adequately removed (Jacob and Wood, 1967; Wong et al., 2025).

SFD formulations enabled the generation of amorphous aprepitant powder over the tested range of solid content, where drug-to-excipient ratios of 20/80 showed promising long-term stability at 25 °C, especially HPC-UL, which exhibited slight superiority in related stability tests (Kožák et al., 2025).

Increasing the solid content from 5% to 10% led to a decrease in specific surface area, but did not affect the initial dissolution rate across all drug-to-polymer ratios. Similarly, contact angle measurements showed only minor trends between the various HPC-based formulations, including physical mixtures.

While distinct levels of supersaturation were reported for HPC-aprepitant formulations in an acidic milieu (pH 1.2) (Kožák et al., 2025), only a slight tendency for supersaturation was observed with HPC-SSL at pH 7.0. To obtain biorelevant and discriminative dissolution profiles, dissolution testing was performed under non-sink conditions. For poorly water-soluble drugs, dissolution under sink conditions can lead to artificially rapid and non-discriminative profiles, whereas non-sink conditions approaching saturation solubility are recommended, particularly for amorphous formulations capable of generating supersaturation (Liu et al., 2013; Trasi et al., 2019). In the nasal cavity, the small volume of mucus and the limited fluid available for drug dissolution mean that intranasal administration naturally occurs under non-sink conditions, where drug dissolution proceeds in a nearly saturated environment (Inoue et al., 2022).

In SFD and film-cast samples, *in vitro* dissolution profiles were influenced by the HPC grade, whereas the physical mixtures showed only minor differences between HPC-UL and HPC-SSL. This suggests that the amorphous nature of the formulation plays a more important role than particle porosity for the total drug amount dissolved *in vitro*. However, during the initial phase of *in vitro* dissolution, SFDs dissolved generally faster than film-cast ASDs. Furthermore, a higher drug load (20% *vs.* 40%) led to a distinct delay in dissolution. Accordingly, an optimum drug-to-polymer ratio would allow high total drug loads while still enabling rapid dissolution.

**Fig. 10:**
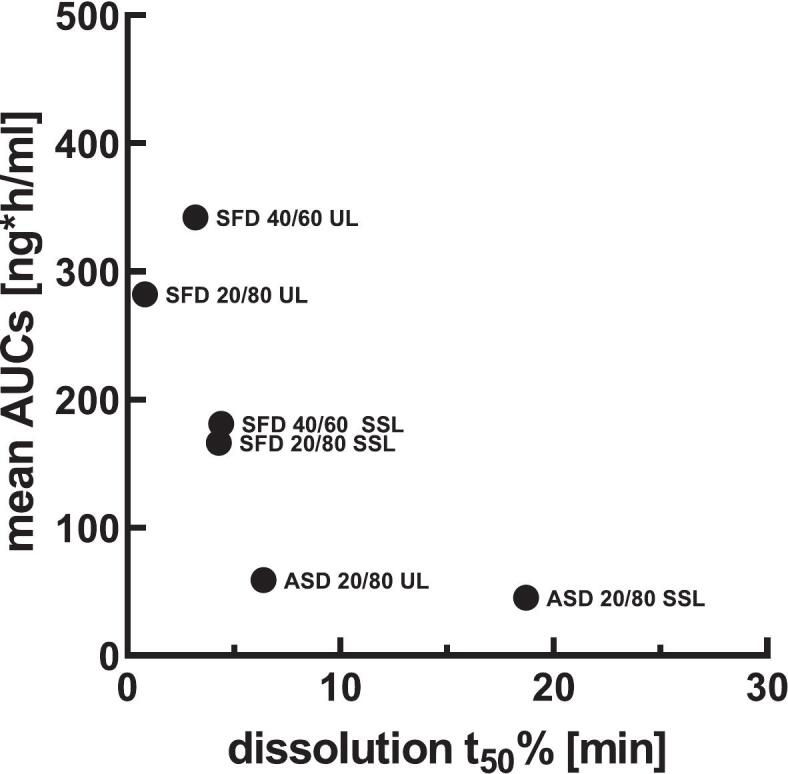
AUC values plotted against *t*₅₀ % from *in vitro* dissolution tests. Data points form three clusters, although the overall correlation between both parameters is limited. Data are presented as mean (*n* = 3); error bars are omitted for clarity.

Conversely, this was only partially reflected in the *in vivo* results, where the film-cast samples led to low bioavailability compared to SFD formulations. *In vitro* saturation solubility showed no impact on *in vivo* performance; however, a trend between accelerated *in vitro* dissolution and increased *in vivo* nasal bioavailability was observed. A correlation between the time required to dissolve 50% of the respective *c*ₛₐₜ *in vitro* (*t*₅₀_%_) and the corresponding mean AUC measured *in vivo* confirms this general trend (Fig. 10). This correlation also summarizes the effects of different formulation parameters on *in vitro* and *in vivo* behavior, with polymer type having the strongest impact (HPC-UL > HPC-SSL), followed by drug form (SFD > film-cast), and drug ratio (20/80 > 40/60).

Initial indications suggest that HPCs can positively influence nasal drug absorption by modulating residence time, although in this earlier study different drugs were administered in their crystalline forms (Tanaka et al., 2020). However, the relatively small group size and interindividual variability should be considered when interpreting these findings. A full understanding of the underlying mechanisms will require further in-depth studies on SFD bioadhesion and swelling behavior on nasal mucosal surfaces, particularly regarding their effects on mucociliary clearance (Merkus et al., 1998; Schipper et al., 1991).

Initial attempts to design freeze-dried particles for nasal administration were based on classical freeze-drying of a budesonide/Soluplus® mixture solubilized in methanol (Pozzoli et al., 2017). The freeze-dried bulk material was subsequently mixed with calcium carbonate to prepare a nasal powder formulation. Although budesonide was present in an amorphous state, an additional bulk mixing step was required to break down the lyophilizate structure, thereby introducing segregation risks in the binary powder mixture due to large density differences. In addition, grinding of the freeze-dried “cake” resulted in broad particle size distributions, increasing the likelihood of smaller fragments reaching the deeper respiratory tract. High amounts of surfactants were also required in this approach.

An alternative solubilization strategy for nasal delivery of poorly water-soluble drugs involved trapping melatonin in cyclodextrins prior to spray-freeze-drying from aqueous solution (You et al., 2024), However, the use of cyclodextrins in the nasal epithelium remains problematic, as it has been repeatedly reported to impair ciliary movement at similar concentrations (Merkus et al., 1999). In another recent study, favipiravir was spray-freeze-dried as favipiravir-pyridinecarboxamide cocrystals from aqueous solution (Wong et al., 2024). Due to the relatively small, flake-shaped particles, approximately 6% of the administered dose reached the lungs, thereby reducing the selectivity of the nasal delivery approach.

Future research on powder formulations for poorly water-soluble drugs intended for nasal administration should focus on identifying an optimum between diametrically opposed requirements, *i.e.*, a particle diameter large enough to avoid accidental pulmonary administration and sufficiently small to ensure complete dissolution and absorption before ciliary elimination (Tiozzo Fasiolo et al., 2018). The relatively large diameter of SFD particles minimizes the risk of unintentional drug delivery to the lungs, since particles with an aerodynamic diameter of around 10 μm or lower are not present in such nasal SFD powders. The use of relatively large particles in combination with amorphous, fast-dissolving aprepitant formulations is therefore particularly promising, as it helps to overcome the drug's poor water solubility.

## 5 Conclusion

Spray-freeze-drying is a promising platform for the nasal administration of poorly water-soluble drugs. Low-viscosity hydroxypropyl cellulose grades, HPC-SSL and HPC-UL, in combination with dimethyl sulfoxide as solvent, enabled the formulation of mechanically stable, porous particles that incorporated and stabilized aprepitant in its amorphous state.

The resulting porous, low-density SFD particles exhibited accelerated *in vitro* drug dissolution compared to physical mixtures of identical composition. *In vivo* studies demonstrated increased bioavailability relative to film-cast formulations. This effect is likely associated with increased porosity and reduced particle density, which may enhance adhesion to the nasal mucosal epithelium and support drug absorption.

Due to its versatility, SFD represents a valuable formulation platform for small, poorly water-soluble molecules that are otherwise difficult to process. Furthermore, this approach may help overcome formulation-related limitations by enabling adaptable dosage forms suitable for multiple routes of administration.

## CRediT authorship contribution statement

**Annika Rautenberg:** Writing – review & editing, Investigation, Data curation. **Paul Bühlbecker:** Writing – review & editing, Investigation, Data curation. **Jan Kožák:** Methodology, Investigation, Data curation. **Claire Chrétien:** Methodology, Investigation. **Yann Pellequer:** Writing – review & editing, Investigation. **Klaus Wunderling:** Writing – review & editing, Investigation, Data curation. **Edmont Stoyanov:** Writing – review & editing, Resources, Conceptualization. **Alexander Pfeifer:** Writing – review & editing, Supervision, Formal analysis. **Alf Lamprecht:** Writing – review & editing, Supervision, Resources, Conceptualization.

## Funding statement

This work was funded and supported by Nippon Soda Co., Ltd., Tokyo, Japan. The University of Bonn and Nisso Chemical Europe GmbH contributed to study design, data collection, analysis, interpretation, manuscript writing, and approval of the final publication.

Edmont Stoyanov is an employee of Nisso Chemical Europe GmbH and does not hold stock in Nippon Soda Co., Ltd. Paul Bühlbecker is a PhD student, Annika Rautenberg and Jan Kozak are postdoctoral fellows, and Alf Lamprecht is a professor at the Department of Pharmaceutics, University of Bonn, Germany. Klaus Wunderling and Alexander Pfeifer are a postdoctoral fellow and professor, respectively, at the Institute of Pharmacology, University of Bonn. Claire Chrétien is a laboratory technician and Yann Pellequer is a professor at the Université Marie et Louis Pasteur (INSERM UMR 1098), Besançon, France. No additional conflicts of interest are declared.

## Declaration of competing interest

The authors declare the following financial interests/personal relationships which may be considered as potential competing interests:

Alf Lamprecht reports financial support was provided by Nippon Soda Co Ltd. Edmont Stoyanov reports a relationship with Nisso Chemical Europe that includes: employment. If there are other authors, they declare that they have no known competing financial interests or personal relationships that could have appeared to influence the work reported in this paper.

## Data Availability

Data will be made available on request.

## Appendix A Supplementary data

Figure S1: Pharmacokinetic data for the different formulations in individual rats (n = 3–4).
