# Supplementary material for: Spray-freeze-drying aprepitant with hydroxypropyl cellulose increases nasal bioavailability
Source: Int J Pharm X. 2026 Apr 26;11:100551. doi: 10.1016/j.ijpx.2026.100551 (PMC13156760; doi:10.1016/j.ijpx.2026.100551)
Supplement: Supplementary file 1 — Figure S1: Pharmacokinetic data for the different formulations in individual rats (n = 3–4). [file mmc1.docx]

**Supplementary information**

Figure S1: Pharmacokinetic data for the different formulations in individual rats (n = 3–4).
